# A Monocular Visual Odometry Method Based on Virtual-Real Hybrid Map in Low-Texture Outdoor Environment

**DOI:** 10.3390/s21103394

**Published:** 2021-05-13

**Authors:** Xiuchuan Xie, Tao Yang, Yajia Ning, Fangbing Zhang, Yanning Zhang

**Affiliations:** National Engineering Laboratory for Integrated Aero-Space-Ground-Ocean Big Data Application Technology, School of Computer Science, Northwestern Polytechnical University, Xi’an 710072, China; xcxie@mail.nwpu.edu.cn (X.X.); yjning@mail.nwpu.edu.cn (Y.N.); fangbing_zhang@mail.nwpu.edu.cn (F.Z.); ynzhang@nwpu.edu.cn (Y.Z.)

**Keywords:** visual odometry, simultaneous localization and mapping, low-texture environment, line segments

## Abstract

With the extensive application of robots, such as unmanned aerial vehicle (UAV) in exploring unknown environments, visual odometry (VO) algorithms have played an increasingly important role. The environments are diverse, not always textured, or low-textured with insufficient features, making them challenging for mainstream VO. However, for low-texture environment, due to the structural characteristics of man-made scene, the lines are usually abundant. In this paper, we propose a virtual-real hybrid map based monocular visual odometry algorithm. The core idea is that we reprocess line segment features to generate the virtual intersection matching points, which can be used to build the virtual map. Introducing virtual map can improve the stability of the visual odometry algorithm in low-texture environment. Specifically, we first combine unparallel matched line segments to generate virtual intersection matching points, then, based on the virtual intersection matching points, we triangulate to get a virtual map, combined with the real map built upon the ordinary point features to form a virtual-real hybrid 3D map. Finally, using the hybrid map, the continuous camera pose estimation can be solved. Extensive experimental results have demonstrated the robustness and effectiveness of the proposed method in various low-texture scenes.

## 1. Introduction

In recent years, intelligent robots have been widely developed and deployed. For instance, as a kind of robot, unmanned aerial vehicle (UAV) has been widely used for resource surveys [1,2], terrain modeling [3], disaster rescue [4] and other applications. For robots, visual odometry (VO) and visual Simultaneous Localization and Mapping (vSLAM) are the key technologies. With visual odometry, a robot can accurately estimate its trajectory in an unknown environment, which endows it with the ability to explore unknown environments on its own [5,6,7,8]. However, possible environments are highly diverse, not always textured, and there are plenty of low-texture environments, such as the streets in urban areas and artificial squares. In these environments, there are no rich textures, but lines are much more abundant due to the structure of the man-made scene. These low-texture environments create unique challenges for VO algorithms.

The VO algorithm is to estimate the motion of the robot using images, which is an important part of vSLAM technology. VO and vSLAM methods are usually divided into two categories: **direct methods** and **feature-based methods**.

The core of the **direct method** is to minimize the photometric errors. Since the direct method only requires the image to have a sufficient image gradient, with no need for feature points, the direct method can be applied to low-texture scenes. In order to avoid the high computation requirement, the direct methods generally exploit only pixels with strong gradients, such as LSD-SLAM [9], a semi-dense direct approach that minimizes the photometric error in image regions with high gradient. Engel et al. [10] proposed a novel direct sparse visual odometry formulation (DSO), which builds a fully direct probabilistic model minimizing the photometric error and implemented the joint optimization of all model parameters, including inverse depth and camera motion. Gao et al. [11] presented LDSO, which is an extension of DSO algorithm to a monocular visual SLAM algorithm featuring loop closure detection and pose-graph optimization. Although these methods can be used in low-texture environments, they worked well in indoor environments. This is because direct methods are based on the grayscale invariant hypothesis, which is a strong assumption that is difficult to guarantee in an outdoor environment. For example, UAVs usually pass through differently lit areas during flight. In addition, direct methods entirely rely on a gradient descent search. Thus, the direct method is valid only if the interframe motion is small, in order to avoid strong nonconvexity of the image gradient. Therefore, direct methods are not suitable for practical large-scale environments. whether it is a low-texture environment or not.

The classic feature-based method is based on **point features**. The core of the feature-based method is to minimize the reprojection error with feature points. In the development of feature point methods, Parallel Tracking and Mapping (PTAM) [12], Stereo Parallel Tracking and Mapping (S-PTAM) [13], and ORB-SLAM2 [14] are the representative works. PTAM is the first representative system for feature-based methods in the keyframe-based SLAM field, which is divided into two separate threads: track and map threads. In track part, a four-level image pyramid is constructed for each frame, and the corner detector is run on each pyramid level. PTAM established the classic framework, followed by S-PTAM and ORB-SLAM2. S-PTAM is a stereo SLAM algorithm with the same framework as PTAM, which used the Binary Robust Invariant Scalable Keypoints (BRISK) extractor [15] to describe features. In contrast to PTAM and S-PTAM, ORB-SLAM2 added a loop closing thread, which made it more complete. ORB-SLAM2 used the Oriented FAST and Rotated BRIEF (ORB) keypoints [16] as point features, and the latest version had been updated to ORB-SLAM3 [17]. However, the performance of the point feature-based methods are closely related to the richness of explicit corner features in the image. Therefore, the performance of the above mentioned methods usually decreases in low-texture environments [18]. In addition, feature matching in low-texture environments is not reliable. Therefore, methods based on point features cannot perform well in low-texture environments.

Despite the lack of reliable feature points, low-texture environments may still contain a number of lines which can be used. Therefore, some researchers have focused on the research based on **line features**. Eade et al. [19] considered line features can provide useful geometric information in structured environments and introduced a concept of edgelet, which consists of a three-dimensional point *x* corresponding with the center of the edgelet, and a three-dimensional unit vector $\hat{d}$ describing the direction of the edgelet. Based on these edgelets, a particle filter framework SLAM was implemented. Zhou et al. [20] proposed the structure line concept, which is represented by a point on a parameter plane and the related dominant direction. Based on the structure line, an extended Kalman filter (EKF) framework SLAM was implemented. In addition, some researchers have utilized both point and line features together in the algorithm. Building upon the ORB-SLAM framework, Pumarola et al. [18] proposed PL-SLAM, a monocular solution which can simultaneously leverage point and line information. In PL-SLAM, lines were parameterized by their endpoints, as suggested by [21], according to the line reprojection error which was defined as the sum of point-to-line distances between the projected line segment endpoints and the detected line in the image plane, the line projection model was constructed. In PL-SLAM, point features and line features were used to construct point and line maps, respectively. Both point and line maps participated in pose optimization. Gomez-Ojeda et al. [22] proposed STVO-PL, a stereo VO approach that combines both points and line segments. The camera motion was also recovered through nonlinear minimization of the projection errors of both point and line segment features. Building upon STVO-PL, Gomez-Ojeda et al. [23] proposed stereo PL-SLAM, a stereo visual SLAM algorithm. For stereo PL-SLAM, a novel bag-of-words approach was proposed, which employs points and line segments to enhance the loop closure process. These methods analyze the lines purely, consider how to parameterize 3D lines and build an error model between 3D lines and observations. Therefore, these methods do not consider the line information utilization from the perspective of feature combination. Furthermore, a significant drawback of using lines directly is that lines are often incompletely detected or partially occluded, leading to instability of the related algorithms. In this paper, by exploring the properties of line segments, we deal with the line segment information from the perspective of combining line segments, that is a novel way to deal with line segments. In this way, many benefits for VO algorithms can be obtained, as elaborated later in the paper.

Although some methods aiming at low-texture environments have been proposed, these methods are mainly for indoor environments. Yang et al. [24] used a single image pop-up plane model [25] to generate plane landmark measurements. Based on the plane landmarks, a plane SLAM algorithm incorporating scene layout understanding was proposed. This approach was designed specifically for corridor scenes and, thus, is limited in scope. In addition, some methods use RGBD camera as sensor. Fu et al. [26] presented a RGBD SLAM algorithm using points and lines for indoor mobile robot pose estimation and 3D map generation. Fabian et al. [27] considered that depth maps can increase the stability in poorly textured environments and that edges are more stable than the raw intensity under different illumination conditions and, thus presented a RGBD VO algorithm that uses a combination of edge images and depth maps to jointly minimize the edge distance and point-to-plane error. Due to the use of a RGBD camera, these methods can only be used in indoor environments.

**Figure 1:**
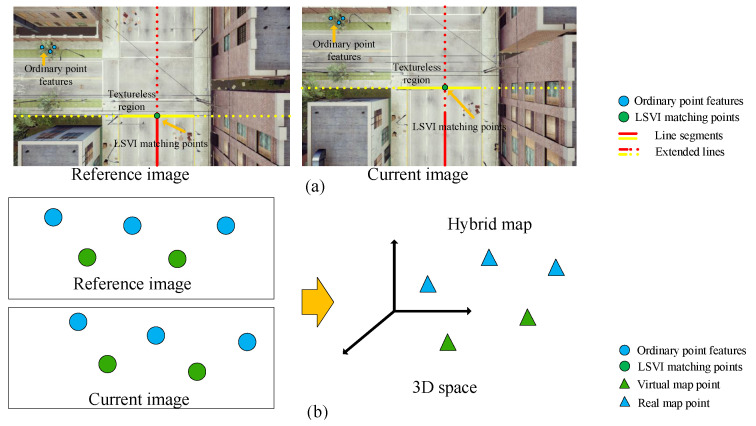
(**a**) The distribution of the LSVI matching points and ordinary point features. The solid lines represent the line segments, the dashed lines represent the corresponding extended lines. The blue and green dots denote ordinary point features and LSVI matching points, respectively. (**b**) The composition of the hybrid map built on the LSVI matching points and ordinary point features. The blue and green triangles represent real and virtual 3D map points, respectively.

For outdoor low-texture environments, only use point features for VO algorithm maybe not sufficient, a feasible solution is to use extra features such as line segment for a complement. Therefore, how to use the line segment information effectively and to propose the corresponding VO algorithm is the goal of this paper. In this paper, we utilize line information from the perspective of combining line segments and define a concept of virtual intersection matching points, based on line segments. By exploring the properties of the virtual intersection matching points, we propose a virtual-real hybrid map based monocular VO algorithm combined with points and line segments. Our main contributions are as follows:

- First, we reprocess line segment features by combining line segments and introduce the concept of virtual intersection matching points.
- Second, we propose the concept of a virtual map using the line segment-based virtual intersection matching points and demonstrate that the virtual map can play the same role as the real map constructed by ordinary point features to participate in camera pose estimation.
- Third, we propose a monocular VO algorithm based on the virtual-real hybrid map, as shown in Figure 1b, which is built on virtual intersection matching points, endpoints of line segments, and ordinary point features.

The remainder of the paper is organized as follows: We describe the proposed concepts of virtual intersection matching points and virtual maps in Section 2. Section 3 describes the proposed virtual-real integrated VO algorithm. In Section 4, we describe the experimental results. In Section 5, we discuss the proposed method. Finally, we conclude the paper in Section 6.

## 2. LSVI Matching Points

We reprocess line segments, from the perspective of combination, in order to generate a new kind of matching points, which we call virtual intersection matching points. As they are based on line segments, we abbreviate them as LSVI matching points. As shown in Figure 1a, in contrast to ordinary point features, the LSVI matching points can be distributed in textureless regions.

We first give the definition of LSVI matching points. Then, we explain how the virtual map generated by the LSVI matching points participates in the estimation of camera poses. Then, we geometrically prove that a pair of LSVI matching points is a correspondence pair, and that there is a corresponding 3D point in space. Finally, we describe the significance of introducing LSVI matching points.

### 2.1. Definition

**Figure 2:**
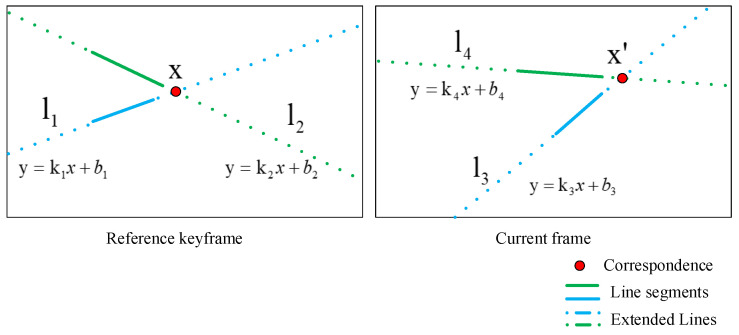
Illustration of the LSVI matching points. The line segments and the corresponding extended lines (shown in the same color) represent the matched line segments and the corresponding extended lines. $k_{m}\left(m=1,2,3,4\right)$ and $b_{n}\left(n=1,2,3,4\right)$ represent the slope and intercept of the line $l_{m}\left(m=1,2,3,4\right)$, respectively. *x* and $x^{\prime }$ represent a pair of correspondences.

As shown in Figure 2, for the current frame image and a reference keyframe image, given two arbitrary pairs of unparallel matched line segments, the matched line segments can produce a virtual point on each of the two images, which is the intersection of the extending lines of the matched line segments. If both virtual points are in the image range, after selection (including epipolar constraint verification and quantitative limitation), a pair of LSVI matching points may be generated. Epipolar constraint [28] is a term in multiview geometry that describes the constraint relationship of a pair of correspondence in two images.

### 2.2. Virtual Map

We explain the virtual map and discuss the role of the virtual map in camera pose estimation.

**Figure 3:**
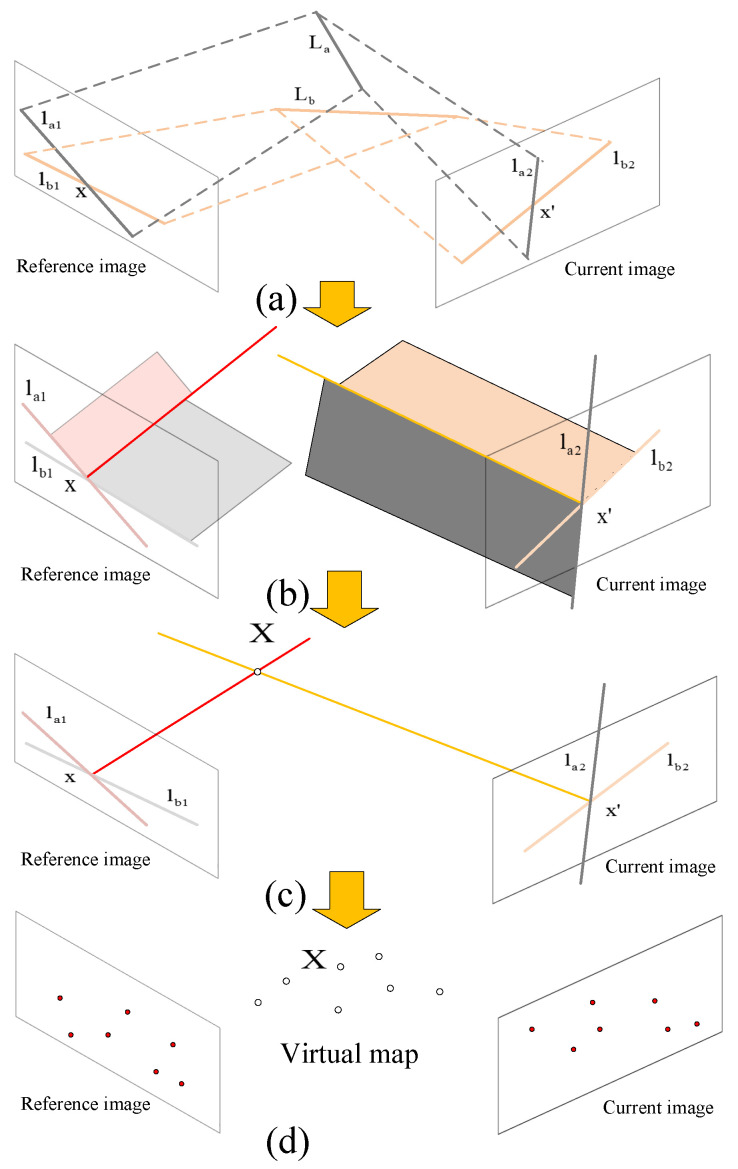
Illustration of the relationship between the matched line segments and the virtual map. The process of generating a virtual map consists of four stages. (**a**) Lines in space are projected onto reference image and current image to get matched line segments. (**b**) Matched lines on image are back projected into space to get two virtual intersection lines. (**c**) Two virtual intersection lines intersect in space to get a virtual 3D point. (**d**) A large number of virtual 3D points constitute a virtual map.

As shown in Figure 3, two unrelated lines $L_{a}$, $L_{b}$ in space are projected onto the reference image through the camera to produce two lines $l_{a1}$, $l_{b1}$. From the knowledge of multiview geometry [28], we can know when a line on an image is back-projected into space, a plane determined by the camera center and the line on the image is obtained. There exists a special line on the plane obtained from the back-projection of line $l_{a1}$; the special meaning is that it also lies exactly on the plane obtained from the back-projection of line $l_{b1}$. In other words, it is an intersection line of the two planes, which is a virtual line that does not exist in real space. Similarly, these two lines $L_{a}$, $L_{b}$ in space can also be used to obtain a virtual line through the process of projection onto the current image and the back-projection into space. The two virtual lines intersect in space to obtain the virtual 3D point *X*. The whole process can be summarized as two unrelated lines in space being used to produce a virtual 3D point through participation of the camera. The projections of the virtual 3D point on two images is a pair of LSVI matching points. A large number of virtual 3D points constitute a virtual map. The structure of virtual maps may be quite different from that of the real world, because they are regenerated from line information, which do not exist in the real world.

The essence of estimating the robot self-motion is to estimate the epipolar geometry (the internal projective relationship between two views) between adjacent images continuously. According to the knowledge in multiview geometry [28], the epipolar geometry is irrelevant to the structure of the scene; it is determined by the motion of the camera itself. We just use the scene structure to estimate the camera trajectory, whether the scene is a mountain or a city street. From this perspective, we can also estimate the camera trajectory with the help of the virtual map as the scene structure.

### 2.3. Demonstration

We give a short proof that, given two pairs of matched line segments on two images, after epipolar constraint verification, a correspondence pair and a corresponding 3D point in space can be determined.

**Figure 4:**
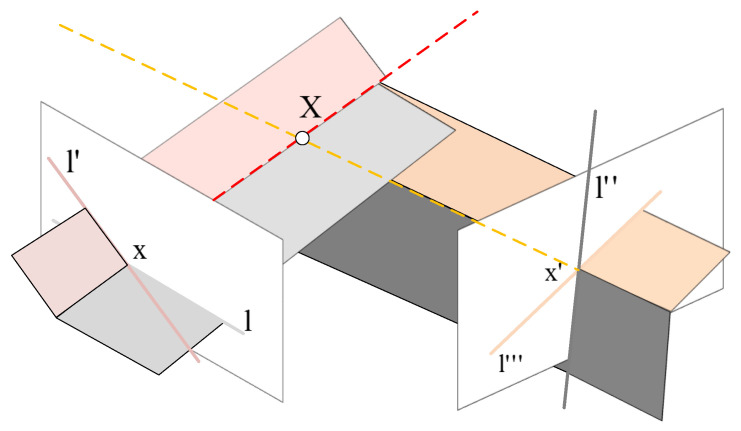
Illustration of the geometrical property of LSVI matching points. There are two pairs of matched line segments on two images (represented by different colors) and four planes determined by these four line segments in space (represented by different colors). *X* represents the 3D point intersected by these four planes.

Obviously, the intersection points of two pairs of matched lines can be regarded as a correspondence pair, as the matching of line segments is unique and two (nonparallel) lines on a plane must meet at one point. We mainly prove that there exists a 3D point corresponding to them. We express the problem in mathematical form. First, each line segment is extended into a straight line on the image plane, since the back projection of a line on an image into space is a plane. In this way, the problem is transformed into whether four planes—determined by four matching lines in two images—intersect in space, as shown in Figure 4. If four planes intersect in space, we prove that there exists a unique 3D point in space.

It should be pointed out that, in this proof, tensor notation is used, where the convention is consistent with that of [28]. We assume that a line on the image is represented by a covariant vector $l_{i}$, and the condition that a image point *x* is on this line is $l_{i}x^{i}=0$. In addition, its corresponding 3D point $X^{j}$ is projected onto the image, such that we can obtain $x^{i}=a_{j}^{i}X^{j}$, where $a_{j}^{i}$ represents the camera projection matrix. Therefore, the condition that a 3D point $X^{j}$ is projected onto the image on the line $l_{i}$ is

(1) $$\begin{array}{c} l_{i}a_{j}^{i}X^{j}=0. \end{array}$$

Therefore, $l_{i}a_{j}^{i}$ represents the plane consisting of all spatial points which can project onto the image on the line $l_{i}$. In order to simplify the description, the four lines are expressed as $l_{p}$, $l_{q}^{\prime }$, $l_{r}^{\prime \prime }$, and $l_{s}^{\prime \prime \prime }$. Therefore, the corresponding four planes can be represented as $l_{p}a^{p}$, $l_{q}^{\prime }a^{q}$, $l_{r}^{\prime \prime }b^{r}$ and $l_{s}^{\prime \prime \prime }b^{s}$, where *p*, *q*, *r*, and *s* respectively represent the row index, and the ith row of the projection matrix is denoted as $a^{i}\left(i=p,q\right)$ and $b^{i}\left(i=r,s\right)$. In general, planes in space do not intersect at the same point; the necessary and sufficient condition for them to intersect is that the determinant of the equation is zero. The corresponding equation is as follows:

(2) $$\begin{array}{c} {}[\begin{array}{c} l_{p}a^{p}\\ l_{q}^{\prime }a^{q}\\ l_{r}^{\prime \prime }b^{r}\\ l_{s}^{\prime \prime \prime }b^{s} \end{array}]\;X=0. \end{array}$$

As the determinant is linear with respect to each row, the determinant *A* of the matrix in the left of Equation (2) can be expressed as:

(3) $$\begin{array}{c} A=l_{p}l_{q}^{\prime }l_{r}^{\prime \prime }l_{s}^{\prime \prime \prime }det\;[\begin{array}{c} a^{p}\\ a^{q}\\ b^{r}\\ b^{s} \end{array}]. \end{array}$$

Then, we use *B* to denote the result of multiplying the determinant *A* times $\left(\varepsilon_{ipq}\varepsilon^{ipq}\right)\left(\varepsilon_{jrs}\varepsilon^{irs}\right)$. As $\varepsilon_{ipq}\varepsilon^{ipq}$ is a scalar value, we recombine it as:

(4) $$\begin{array}{c} B=\left(l_{p}l_{q}^{\prime }\varepsilon_{ipq}\right)\left(l_{r}^{\prime \prime }l_{s}^{\prime \prime \prime }\varepsilon_{jrs}\right)\varepsilon^{ipq}\varepsilon^{irs}det\;[\begin{array}{c} a^{p}\\ a^{q}\\ b^{r}\\ b^{s} \end{array}]. \end{array}$$

As the expression $\left(l_{p}l_{q}^{\prime }\varepsilon^{ipq}\right)$ is the cross product of the two lines $l_{p}$ and $l_{q}^{\prime }$–it is their intersection $x^{i}$ [28]—in the same way, the expression $\left(l_{r}^{\prime \prime }l_{s}^{\prime \prime \prime }\varepsilon^{jrs}\right)$ is equal to the intersection $x^{\prime j}$. Therefore, Equation (4) can be expressed as:

(5) $$\begin{array}{c} B=x^{i}x^{\prime j}\varepsilon_{ipq}\varepsilon_{irs}det\;[\begin{array}{c} a^{p}\\ a^{q}\\ b^{r}\\ b^{s} \end{array}]. \end{array}$$

As the epipolar constraint is satisfied, the properties of the fundamental matrix can be used. By means of a result about the fundamental matrix presented in [28], the determinant $F_{ji}$ of the fundamental matrix can be represented as:

(6) $$\begin{array}{c} F_{ji}=\left(1/4\right)\varepsilon_{ipq}\varepsilon_{irs}det\;[\begin{array}{c} a^{p}\\ a^{q}\\ b^{r}\\ b^{s} \end{array}]. \end{array}$$

When Equation (6) is substituted into Equation (5), we obtain

(7) $$\begin{array}{c} B=4x^{i}x^{\prime j}F_{ji}. \end{array}$$

As one of the basic properties of the fundamental matrix is that its determinant is zero, from the above derivation, we have

(8) $$\begin{array}{c} A=l_{p}l_{q}^{\prime }l_{r}^{\prime \prime }l_{s}^{\prime \prime \prime }det\;[\begin{array}{c} a^{p}\\ a^{q}\\ b^{r}\\ b^{s} \end{array}]\overset{def}{=}x^{i}x^{\prime j}F_{ji}=0. \end{array}$$

As the determinant *A* is zero, that the four planes determined by the four matching lines on the two images intersect in space is proved. Therefore, given two pairs of matched line segments on two images, a 3D point can be determined in space. For the details of the proof and tensor notation, refer to [28].

### 2.4. Significance of Introducing LSVI Matching Points

**Figure 5:**
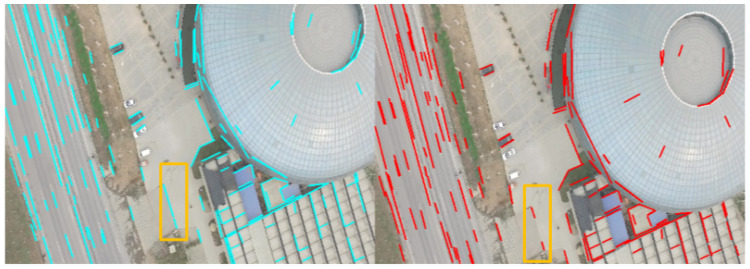
Illustration of the incomplete detection of line segments. The short lines with cyan and red color represent line segments in the two images.

First, LSVI matching points cannot be affected by inconsistent line segment lengths. In the process of line segment matching, two wrong line segments with different lengths may be matched. The case is due to the occlusion or incomplete detection of a line segment, as shown in Figure 5, although line segments in orange box are in the correct direction, due to the incomplete detection of the line segments, the endpoint matching of these line segments is wrong. However, the LSVI matching points are not affected by the inconsistent line segment lengths, as the construction of LSVI matching points is determined by the position and direction of the line, not by the length of the line segment.

**Figure 6:**
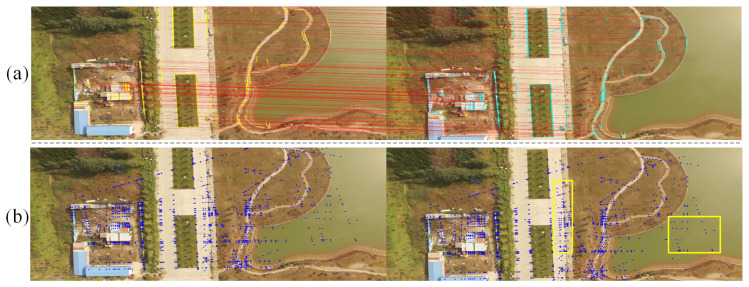
Illustration of the distribution of the LSVI matching points. (**a**) Matched line segments. The matched line segments are connected by red lines, and the matched line segments are represented by yellow and cyan short lines, respectively. (**b**) LSVI matching points. The LSVI matching points are represented by blue dots.

Second, the generation of the LSVI matching points essentially involves the reprocessing of line segment information, such that the processing result significantly increases the number of features and makes the distribution of feature points more uniform. Figure 6a shows the matched line segments in two images, and the LSVI matching points generated from matched line segments are shown in Figure 6b. As shown in yellow rectangles in Figure 6b, the LSVI matching points can be distributed in places where there would not have been ordinary feature points, such as on lakes and asphalt pavement. We know that Perspective-n-Point (PnP) [29] is a key step in vSLAM or VO algorithms, which is used to solve the initial camera pose of the current frame. The accuracy of PnP depends on the number of observations and their distribution in the image [29]. A large number of 2D–3D correspondences provide redundancy for estimation, while a uniform distribution of points avoids bad-conditioned configuration [30]. For this reason, ref. [30] proposed a score strategy, which aims to select those images with uniform distribution and more observations to participate in the reconstruction. In ORB-SLAM2, in order to make the extracted features more dispersed, the image is divided into root grids, in which features are extracted. Then, using quadtree, each grid is divided into four parts each time; once the node has only one key point or the number of key points has met the requirements, the segmentation is stopped. Finally, the point with maximum Harris response in each node is stored. Compared with using only the endpoints of line segments, the use of LSVI matching points increases the number of features and makes the distribution of features more uniform, which is beneficial for the PnP algorithm, such that a more accurate initial camera pose estimate can be obtained.

Third, in vSLAM or VO algorithms, in order to obtain a more accurate camera pose estimate, local optimization through bundle adjustment (BA) [31] is necessary. Obviously, with the use of the virtual map, the amount of data is increased, which can lead to better optimization results.

## 3. Virtual-Real Integrated VO Algorithm

**Figure 7:**
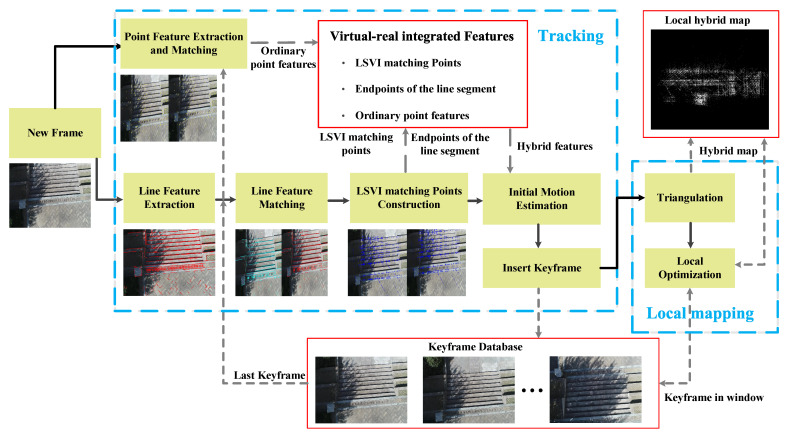
Framework of the proposed virtual-real hybrid map based monocular VO algorithm. The algorithm includes two threads: tracking and local mapping. The black arrows denote the connection of steps and the gray dashed lines denote the data transfer in different steps.

The proposed virtual-real hybrid map based monocular VO algorithm is illustrated in Figure 7. When processing a new frame, the point and line segment features are first extracted and matched. According to the matched line segments, the LSVI matching points are constructed. Then, according to the processed data, the initial motion estimation for current frame pose and the decision of whether to insert a keyframe are performed. Once a keyframe is inserted, the local mapping thread creates the hybrid map and performs local optimization to obtain a better estimate. The VO algorithm follows the classic keyframe-based vSLAM framework, including two threads, tracking and local mapping; however, it does not include a loop closing thread. The key parts of the algorithm, including the construction of the LSVI matching points and the management of frames and maps, are described in detail as follows.

### 3.1. LSVI Matching Points Construction

**Figure 8:**
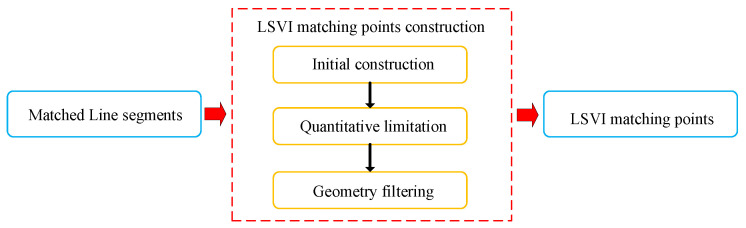
A flowchart of constructing LSVI matching points. The input of the algorithm module is the matched line segments, and the output is the LSVI matching points.

The construction of the LSVI matching points is based on the processing of line segments. The main steps are shown in Figure 8. To extract line segments, the Line Segment Detector (LSD) [32] was employed, which is fast and provides high precision. Before feature matching, we use the Line Band Descriptor (LBD) method [33] as the binary descriptor to augment line segments. Then, we match the line segment features detected in two images. After line segment matching, geometric filtering is required, in order to get rid of mismatches. As a line segment has two endpoints, we perform double geometric filtering. For matched line segments, the two endpoints of a line segment are respectively stored in two independent endpoint sets. Then, we perform geometric validation by constructing a fundamental matrix for the two endpoint sets. For each pair of matched line segments in the two images, if both endpoints pass their respective geometric verification, this pair of matched line segments is retained.

The construction of the LSVI matching points includes two steps: initial construction and subsequent selection. In the initial construction, line segments in the reference keyframe are divided into $N_{g}$ groups (e.g., 36 groups), according to their directions. Line segments are assigned by direction, some groups may have multiple line segments, while other groups may not have any line segments. We randomly take two nonparallel line segments from different groups and extend them into straight lines according to the image coordinates of their endpoints. Lines are expressed in slope–intercept form, and an intersection point is calculated by solving the equations of the two lines jointly, as shown in Figure 2. If the coordinate of the intersection point is in the image range, it is valid and will be retained. In the same way, using two line segments of corresponding index in the current frame, the coordinate of an intersection point are calculated. If it is valid, a pair of initial LSVI matching points is obtained. The initial construction of the LSVI matching points is completed after traversing line segments in all groups.

The selection step includes two parts: In the first part, we limit the number of matching points. In the second part, we perform geometric verification using the fundamental matrix. Given *N* line segments, in the extreme case, at most N∗(N−1)/2 LSVI matching points will be generated. In fact, the number of LSVI matching points cannot be as large as N∗(N−1)/2, as line segments from the same group cannot produce LSVI matching points, and a pair of LSVI matching points is regarded as valid only if both of them are located in the image area of two frames, respectively. However, sometimes, the number of LSVI matching points may still be large. A larger number of map points is beneficial to the robustness of the algorithm, but the construction of a large number of map points will restrict the efficiency of the algorithm. Our purpose is to introduce virtual map points to enhance the stability of the algorithm; however, on the other hand, the algorithm efficiency should not be reduced significantly due to constructing too many map points. Therefore, to limit the number of LSVI matching points, we need to select from the initial sets. Inspired by ORB-SLAM2, we divide the reference image into *M* grids and allocate the LSVI matching points into corresponding grids, according to their coordinates. If the number of points in a grid is less than $N_{grid}$, the LSVI matching points in this grid are all preserved. If there are more than $N_{grid}$ points in a grid, the points are sorted according to the response values of line segments constituting these LSVI matching points. At most, $4*N_{grid}$ points with larger response values are reserved. In the second part of the selection, a fundamental matrix is first constructed between reference keyframe and current frame using the endpoints of the matched line segments and ordinary point features. Then, geometric verification is performed to select the LSVI matching points conforming to the epipolar constraint.

### 3.2. Hybrid Map

Three types of points are used: LSVI matching points, endpoints of line segments, and ordinary point features. We use SiftGPU [34], a Graphics Processing Unit (GPU) implementation of the Scale-invariant Feature Transform (SIFT) as the ordinary point feature. We use the Direct Linear Transformation (DLT) algorithm [28] for triangulation, to build the virtual-real integrated hybrid map.

In order to obtain an accurate estimate, local BA is used to optimize the poses in a local window of covisible keyframes and points in the hybrid map. The optimization problem is solved as:

(9) $$\begin{array}{c} \min_{R_{i},t_{i},X_{\left(.\right)}^{j}}\sum_{i\in \kappa }\sum_{j\in \chi }\rho \;\|\;x_{\left(.\right)}^{ij}-\pi \left(R_{i}X_{\left(.\right)}^{j}+t_{i}\right)\;\|, \end{array}$$

where κ represents a group of keyframes; χ represents the local hybrid map; $x_{\left(.\right)}^{ij}$ represents the observation, either an LSVI matching point, an endpoint of the line segment, or an ordinary feature point; $X_{\left(.\right)}^{j}$ represents a corresponding hybrid 3D map point, either a real map point or a virtual map point; $R_{i}$ and $t_{i}$ are the rotation matrix and translation vector of the ith frame, respectively; ρ is the robust Huber cost function; and π is the projection function, defined as follows:

(10) $$\begin{array}{c} \pi ([\begin{array}{c} \begin{array}{c} X\\ Y\\ Z \end{array} \end{array}])=[\begin{array}{c} f_{x}\frac{X}{Z}+c_{x}\\ f_{y}\frac{Y}{Z}+c_{y} \end{array}], \end{array}$$

where $f_{x},f_{y}$ are focal lengths and $\left(c_{x},c_{y}\right)$ is the principal point, all of which are known from calibration. In optimization, the hybrid 3D map point $X_{\left(.\right)}^{j}$, rotation matrix $R_{i}$ and translation vector $t_{i}$ are the variables to be optimized. To solve the optimization defined in Equation (9), we use the Levenberg–Marquardt algorithm [35], implemented in g2o [36], as the solver.

### 3.3. Frame Management

Up to $N_{kf}$ keyframes are kept in a window. Each new frame is initially tracked with respect to a reference keyframe. According to two criteria, the current frame is either discarded or used to create a new keyframe. First, a new keyframe needs to be created when the field of view has changed significantly. We measure the shift of the endpoints of matched line segments by

(11) $$\begin{array}{c} dist=\frac{1}{n}\left(\sum_{i=1}^{n}\|p-p^{\prime }\right){{\|}^{2})}^{\frac{1}{2}}, \end{array}$$

where *n* represents the number of endpoints of matched line segments. If dist is more than 0.2× *h* pixels (*h* is the height of the image), a new keyframe is created. Second, another key factor is the number of matched points. If the number of matched ordinary feature points or LSVI matching points is less than threshold (e.g., 500) a new keyframe is created.

## 4. Results

To evaluate the performance of the proposed virtual-real integrated monocular VO algorithm, we conducted extensive experiments, including qualitative and quantitative experiments. First, we performed a qualitative evaluation to verify the robustness of the proposed method in various low-texture environments using aerial data collected by a DJI Phantom 4. Then, quantitative experiments were carried out to further evaluate the performance of the proposed method using image sequences of simulated environments. The evaluations were carried out on a PC with an Intel Core i7 CPU with 2.8 GHz, 8 GB of RAM and a NVIDIA GeForce GTX 1060 GPU with 6 GB of VRAM.

### 4.1. Qualitative Evaluation

**Figure 9:**
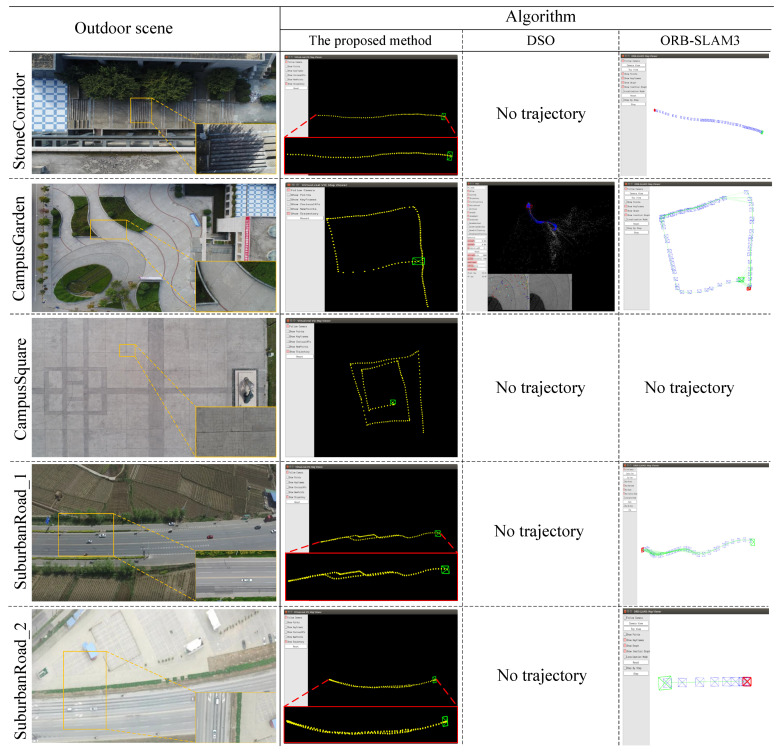
Qualitative comparison experiments of real outdoor scenes. For the results of our method, the yellow dots represent the UAV 3D positions, the green square represents the current pose of the UAV and the “zoom-in” views are shown in the red boxes. For the results of ORB-SLAM3, the blue camera shape pattern represents the pose of the keyframe. For the results of DSO, the green line represents the trajectory and the blue camera shape pattern represents the pose of the keyframe.

We conducted qualitative experiments to validate the robustness of the proposed algorithm in different real low-texture outdoor scenes. Although we cannot get the ground truth of the UAV flight, since we manually controlled the drone to follow a preset flight path, we can know the approximate trajectory of the drone and how long the trajectory is. By analyzing the integrity of the estimated trajectory, the robustness of the different algorithms can be compared. We compared our method against DSO [10] and ORB-SLAM3 [17], which is the representative of direct and feature-based methods, respectively. We used a DJI Phantom 4 to capture aerial videos inside and outside the campus. Before the experiments, we calibrate the camera intrinsic parameters using a calibration tool, Toolbox_calib of Matlab, and then distortion correction is performed according to the distortion parameters calibrated in advance. The scenes included a stone corridor, a campus garden, a campus square, and two suburban roads in different seasons. In the first column of Figure 9, we show a panorama of each scene. A representative aerial video frame is shown at the bottom right of each panorama, and the approximate location where image was captured is marked on the panorama. Due to the influence of manual control and wind force, there was a continuous certain vibration during the flight. In addition, restricted by the sight distance, there was some deviation when flying far away.

The first row of Figure 9 displays a flight track over a stair-stepping stone corridor which was 56 m long and descended 4 m. The UAV moved at 6 m above the highest flat ground and captured a video with resolution of 1280 × 720. The main content of the image was the stone ladder, and a few plants were captured when flying to the bottom of the stone ladder. The comparison results are shown in the right of the first row of Figure 9. Our method could estimate the complete trajectory. In contrast, DSO could not estimate the trajectory because the direct method represented by DSO requires harsh conditions, with small motion and constant illumination. However, the actual data do not meet these conditions. On the one hand, this data set exhibited the significant change in illumination; on the other hand, outdoor drones cannot fly as slowly as robots move indoors, which cause serious challenges to direct methods such as DSO. Due to the insufficient features, ORB-SLAM3 could not perform successful initialization immediately. Initialization refers to the establishment of a reliable initial map from sufficient features and then the start of trajectory estimation. Due to the delay in initialization, the trajectory estimated by ORB-SLAM3 was incomplete, (i.e., the first 12.5% of the sequence was missing). However, our method could perform initialization successfully and rapidly on this data set, since the upper limit of the number of LSVI matching points is very large. In the initialization stage, we temporarily ignored the problem of efficiency and no limit was placed on the number of LSVI matching points; that is, as long as a pair of LSVI matching points was within the image range and conformed to the epipolar constraint, it was considered valid.

As shown in the second row of Figure 9, the drone flew over a campus garden along a 9-shaped path. The content of this scene was relatively rich. In addition to stone materials, there were some green plants. The drone flew 10 m above the ground and captured a video with resolution of 1280 × 720. Our method estimated the complete trajectory and the trajectory looked very smooth, with small fluctuations and an even interval. Although DSO generated part of the trajectory, it was obviously wrong. Again, ORB-SLAM3 could not perform initialization immediately and, thus, an incomplete trajectory is obtained, which looked like a rectangle instead of a 9-shaped path (i.e., the first 23.4% of the sequence was missing).

From the third row of Figure 9, it can be seen that the drone flew over a campus square along a spiral square polyline path. The drone flew 10 m above the ground and captured a video with resolution of 1280 × 720. The content of this scene is very simple, only comprised of stone floor bricks with no patterns; thus, this scene was extremely challenging and both DSO and ORB-SLAM3 could not estimate the trajectory. The reason of failure is that ORB-SLAM3 could not perform initialization successfully. In contrast, our method obtained a complete trajectory.

As shown in the fourth row of Figure 9, the drone made a roundtrip flight along a suburban road outside the campus. In other words, the drone first moved east along the road, then turned back and moved west. In the process of UAV round-trip movement, a part of tracks did not overlap, which was caused by manual control and wind influence. It should be noted that the actual track was longer than that shown in the panorama. The UAV captured a video with resolution of 1920 × 1080. Although the main content of image was asphalt pavement, there was continuous vegetation along both sides of the road and the feature distribution on the vegetation was much denser than on the asphalt pavement. In addition, vehicles sometimes passed on the road, making it a dynamic environment, which further increased the difficulty. ORB-SLAM3 only estimated a fraction of the trajectory for a section of the road with relatively rich feature points; however, for the low-texture fraction of the road, ORB-SLAM3 cannot estimate the trajectory, the reason for which was the initialization delay and lost tracking. Lost tracking refers to the failure of camera pose estimation due to the insufficient feature points in some frames, which makes VO algorithm unable to continue. Therefore, due to the two reasons, for ORB-SLAM3, in total, 55% of the sequence was missing. DSO could not estimate the trajectory. In contrast, our method estimated a complete trajectory.

The fifth row of Figure 9 displays another roundtrip flight along a suburban road outside the campus in winter. It should be noted that the actual flight track was longer than the road section shown in the panorama. Compared with the previous group of experiments, the trees and other vegetation on both sides of the road became withered in winter, such that the features on the vegetation became more rare. In addition, the drone flew over a large artificial construction area, such as a large parking lot. Thus, this scene was even more challenging. Due to the lost tracking and initialization delay, ORB-SLAM3 only estimated a part of the trajectory, missing 93% of the sequence. DSO still could not estimate a trajectory. In contrast, our method estimated a complete trajectory.

From the qualitative evaluation, we can see that the proposed method has good robustness in different real outdoor low-texture scenes.

### 4.2. Quantitative Evaluation

**Figure 10:**
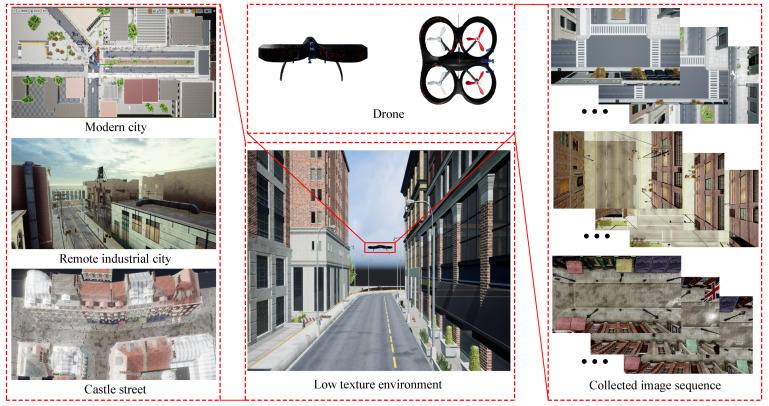
Illustration of collecting images using Airsim. On the left, various scenes are displayed; in the middle, an example of the drone crossing the scene is shown; and, on the right, an example of some collected image sequences is given.

We also conducted quantitative evaluations. Due to a lack of ground truth in the aerial videos collected by the DJI Phantom 4, we used Airsim [37], combined with Unreal Engine, to obtain aerial images with ground truth in various simulation scenes. Airsim is a simulator for drones or vehicles built on Unreal Engine. With this tool, we can simulate the flight of a drone and collect data, including RGB images and GPS values, from Unreal Engine. For the experiment, we adopted various environments, including a modern city, a remote industrial city, and a castle town, as shown in Figure 10.

**Figure 11:**
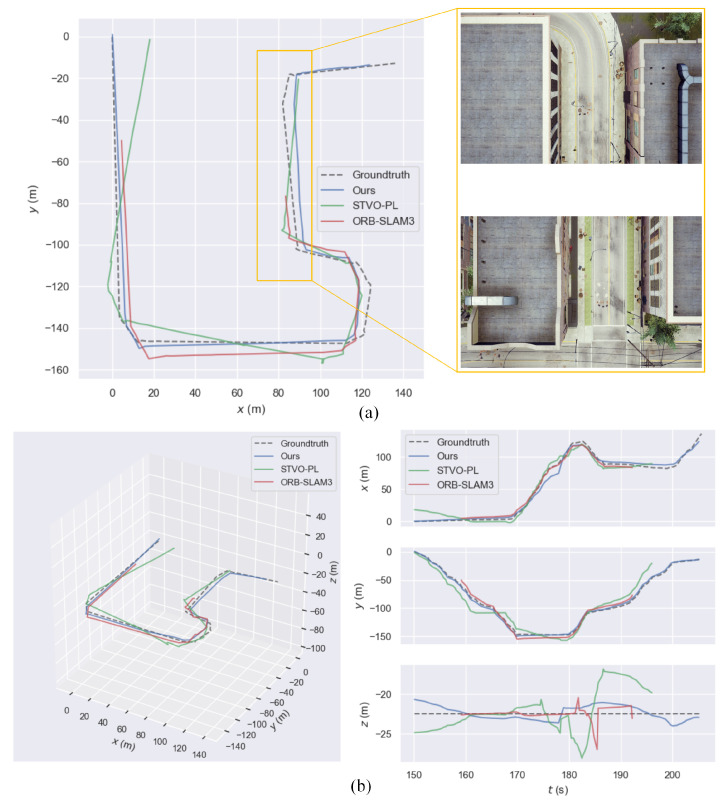
(**a**) Comparison of trajectories against ground truth on the IndustrialCity-downward simulation sequence. (**b**) Comparison of trajectories on the IndustrialCity-downward simulation sequence on xyz three-axis.

In the simulation experiments, there were four image sequences and the resolution of images was 640 × 480. For the castle street scene, two image sequences were collected: Downward-looking and forward-looking. For the modern city and remote industrial city scenes, downward-looking sequences were collected. To make the experiment more challenging, the selected scenes were man-made scenes based on stone materials and the drone moved at a low height; thus, most of the content in downward-looking images was roads, as shown in Figure 10. In order to generate the plots for quantitative evaluation, we used the evo package [38]. Due to the unknown real scale, we adopt the alignment mode of the evo tool to scale and align the trajectories of ORB-SLAM3, STVO-PL, and the proposed method with the ground truth trajectories. STVO-PL is a representative point and line combination VO method. As DSO could not estimate camera trajectories for these image sequences, we could not plot them for comparison. Figure 11a shows a comparison of the computed trajectories, compared to the ground truth. As shown in Figure 11a, for the IndustrialCity-downward sequence, both STVO-PL and ORB-SLAM3 only estimated a part of the trajectory before the tracking was lost. The positions of lost tracking were located in a low-texture road section, where the road was narrow and the roofs on both sides were smooth, while a few facades of buildings were captured while the drone was crossing this road section. As a point and line combination method, the estimated trajectory of STVO-PL was a little longer than that of ORB-SLAM3. In addition, the trajectories obtained by STVO-PL and ORB-SLAM3 showed frequent violent fluctuations. In fact, the drone moved very smoothly in the simulation environment, such that there was no fluctuation in the real trajectory. In contrast, our method showed robust performance and obtained a complete and smooth trajectory. In addition, we use Figure 11b to show the trajectories’ comparison on xyz three-axis. As shown in Figure 11b, the trajectory obtained by the proposed method is closest to the ground truth on xyz three-axis, which shows the good performance of the proposed method. Table 1 shows the absolute RMSE of the different methods. The experimental results indicate that our method can obtain better performances on most sequences, except CastleStreet-f sequence, which was composed of forward-looking images. The content of the image in CastleStreet-f sequence was the horizontal view captured by UAV flying in the street. The ordinary point features distributed on the building facade were relatively rich. Therefore, ORB-SLAM3 can work well in this sequence.

## 5. Discussion

We discuss the combination of ordinary point features and the LSVI matching points.

**Figure 12:**
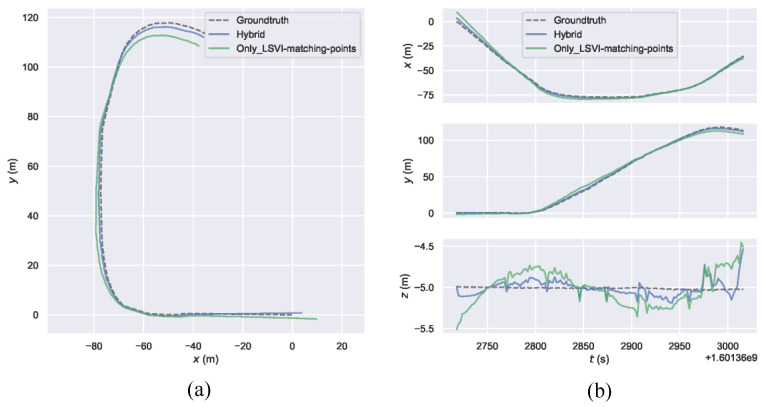
Quantitative comparisons of the algorithm with only LSVI matching points and the algorithm with hybrid features against the ground truth in simulation experiments: (**a**) Trajectory comparisons on ModernCity-downward sequence; and (**b**) Position error comparisons on ModernCity-downward sequence.

Our main reason for using ordinary point features is to ensure the stability of the algorithm in environments with few lines. For scenes with few lines, although the line segment features are few, the algorithm can still run stably due to the rich point features present. For low-texture environments with rich lines, it is feasible to use only LSVI matching points in the VO algorithm. We conducted experiments on outdoor simulation data sets, in order to verify the VO algorithm performance using only LSVI matching points. Figure 12 shows an example on the ModernCity-downward sequence. We can see that the algorithm with only LSVI matching points obtained similar performance as the algorithm using the combination of ordinary point features and LSVI matching points. The performance of the algorithm with hybrid features was better, as the number of features was more abundant and the distribution of features was more uniform. Since our algorithm uses all kinds of features, the application is not limited; on the other hand, our algorithm has the advantage of strong robustness in low-texture environment.

We show the average timing results in Table 2 on the simulation data set. The average processing time of each frame was 56.2 ms for an image with resolution of 640 × 480. The average processing time of ORB-SLAM3 and STVO-PL was 33.1 ms and 47.8 ms, respectively. The algorithm contains two parallel threads: tracking and local mapping. Tracking is executed for every frame, but the local mapping thread does not work all the time. When a new key frame is added, the local mapping thread will complete the triangulation and BA operation. Although the time of mapping is 65.1 ms once, for the total system, the average processing time is 56.2 ms. since we use two kinds of features, line segment and ordinary point. Considering the efficiency, the two kinds of features can be calculated synchronously and independently. However, since the calculation time of line segment features (i.e., 39.1 ms and 2.84 ms) is longer than that of point features (i.e., 25.0 ms and 1.00 ms), the time consumption of feature extraction and matching (i.e., 39.1 ms and 2.84 ms) is basically equal to that of line segment features. Since line segment processing is a time-consuming operation, although the stability of the system in the low-texture environment is improved, after introducing line segment features, the efficiency of the system will decline, which is a limitation of this paper. Note that our method is independent of line feature detection and matching, so it is beyond the scope of this paper to improve their efficiency.

## 6. Conclusions

Different from the traditional methods which only use the real map, generated by ordinary point features to perform visual odometry estimation. In this paper, we defined a concept of LSVI matching points and demonstrated that the virtual map constructed from the LSVI matching points can be used to effectively estimate the camera pose. Based on this idea, we proposed a novel virtual-real hybrid map based monocular visual odometry algorithm. The camera pose estimation was solved through the hybrid 3D map, composed of virtual map generated by LSVI matching points and real map generated by ordinary point features. The proposed method can improve the stability of the VO algorithm under low-texture environment. Extensive experiments were carried out to verify the effectiveness and robustness of the algorithm. For future work, we plan to add a loop closure detection thread to our current VO algorithm, in order to realize a complete virtual-real integrated SLAM algorithm and, thus, eliminate the scale drift.

## Figures and Tables

**Table 1:** Comparison of RMSE (m) on simulation data set. ‘-d’ stands for downward and ‘-f’ stands for forward, ‘x’ indicates no trajectory or too short trajectory using this method.

Methods	IndustrialCity-d	CastleStreet-d	CastleStreet-f	ModernCity-d
ORB-SLAM3	9.501	0.491	0.129	2.124
DSO	x	x	x	x
STVO-PL	13.90	1.787	0.662	4.517
Ours	5.186	0.467	0.199	1.384

**Table 2:** Timing results for each thread on the simulation data set.

Thread	Operation	Time (ms)
Tracking	Feature detection	39.1
	Feature matching	2.84
	LSVI matching points construction	3.98
	Initial motion estimation	4.13
	Total tracking	51.1
Local mapping	Triangulation	13.7
	BA	51.4
	Total mapping	65.1
Total mean		56.2

## Data Availability

Not applicable.

## Abbreviations

The following abbreviations are used in this manuscript:

VO	Visual odometry algorithm
SLAM	Simultaneous Localization and Mapping algorithm
UAV	Unmanned aerial vehicle
LSVI	Line segments based virtual intersection
GPU	Graphics Processing Unit
SIFT	Scale-invariant Feature Transform
DLT	Direct Linear Transformation
PnP	Perspective-n-Point
ORB	Oriented FAST and Rotated BRIEF
BRISK	Binary Robust Invariant Scalable Keypoints
EKF	Extended Kalman filter
PTAM	Parallel Tracking and Mapping
S-PTAM	Stereo Parallel Tracking and Mapping
